# In-hospital cardiac arrest incidence and outcomes in the era of COVID-19: an observational study in a Singapore hospital

**DOI:** 10.1186/s12245-021-00356-7

**Published:** 2021-05-31

**Authors:** Ting Lyu, Faheem Ahmed Khan, Shanaz Matthew Sajeed, Amit Kansal, Monika Gulati Kansal, Shekhar Dhanvijay, Rou An Tan, Jared D’Souza, Ian Cendana, Patricia Leong, Chee Keat Tan

**Affiliations:** 1 Department of Intensive Care Medicine, Ng Teng Fong General Hospital, National University Health System, 1 Jurong East Street 21, Singapore, 609606 Singapore; 2 Department of Intensive Care Medicine, the First Affiliated Hospital, College of Medicine, Zhejiang University, Hangzhou, China

**Keywords:** COVID-19 pandemic, In-hospital cardiac arrest

## Abstract

**Background:**

COVID-19 pandemic has resulted in significant strain on healthcare resources and this requires diligent resource re-allocation. We aim to describe the incidence and outcomes of in-hospital cardiac arrest (IHCA) during this period as compared to non-pandemic period.

**Methods:**

We conducted a retrospective study in a tertiary care hospital in Singapore. The study compared the incidence and outcomes of code blue activations over a 3-month period from March to May 2020 (COVID-19 period) with the same months in 2019 (pre-COVID-19 period). The primary outcome of the study was the rate of survival to hospital discharge for IHCA. The secondary outcomes included incidence of all code blue activation per 1000 hospital admissions, incidence of IHCA per 1000 hospital admissions.

**Outcomes:**

The rate of survival to hospital discharge for IHCA was 5.88% in the COVID-19 period as compared to 10.0% in the pre-COVID-19 period [odds ratio (OR), 0.72; 95% confidence interval (CI), 0.26-1.95]. Compared to pre-COVID-19 period, there were more IHCA incidences per 1000 hospital admissions in the COVID-19 period (1.86 vs 1.03; OR, 1.81; 95% CI, 0.78-4.41).

**Conclusions:**

The study observed a trend towards higher incidence of IHCA and lower rate of survival to hospital discharge during COVID-19 pandemic compared to pre-COVID-19 period.

## Introduction

Coronavirus disease 2019 (COVID-19) pandemic has caused a global healthcare crisis. Till early September 2020, nearly 27 million COVID-19 cases and 900,000 deaths have been reported to the World Health Organisation (WHO) [1]. As a highly transmittable disease, COVID-19 has posed a significant threat to healthcare workers. A report from the Centers for Disease Control and Prevention (CDC) COVID-19 response team showed that 19% of COVID-19 cases were identified as healthcare personnel among 49,370 cases in the USA [2]. In Singapore, the cumulative number of cases has exceeded 57,000 at the time of writing, with 27 cases demised [3]. No healthcare workers have contracted the COVID-19 virus in the workplace with strict precautions in place after lessons from *severe acute respiratory syndrome* (*SARS*) [4].

Ng Teng Fong General Hospital (NTFGH) is a healthcare institution located in the west of Singapore with a novel code blue activation system [5]. So far, NTFGH has admitted over 2000 COVID-19 cases with varying severity. There is a dedicated hospital code blue team (HCBT) to attend to code blue activations in NTFGH. Since late February 2020, with more COVID-19 cases reported in Singapore, HCBT in NTFGH adopted full precautions to handle all code blue cases in order to minimise the risk of COVID-19 exposure [6]. There have been studies describing the increased IHCA incidence with worse outcomes in COVID-19 patients, but few studies are available to evaluate the overall impact of COVID-19 pandemic on incidence and outcomes of IHCA which includes non-COVID-19 patient s[7–10].. Hence, we conducted a retrospective study in our institution to assess the incidence and outcomes of IHCA during this period.

## Methods

### Setting

We conducted this retrospective observational study in NTFGH. In NTFGH, code blue could be activated through the Automated Code Blue Alert and Activation (ACBAA) system or by pressing the code blue button installed on every patient’s bed. The HCBT consisted of an intensive care unit (ICU) consultant, an ICU registrar, an ICU nurse, a medical registrar and a respiratory therapist. Each team member carried an internet proxy phone where the location of the code blue activation would be shown upon activation.

### ACBAA system

**Fig. 1:**
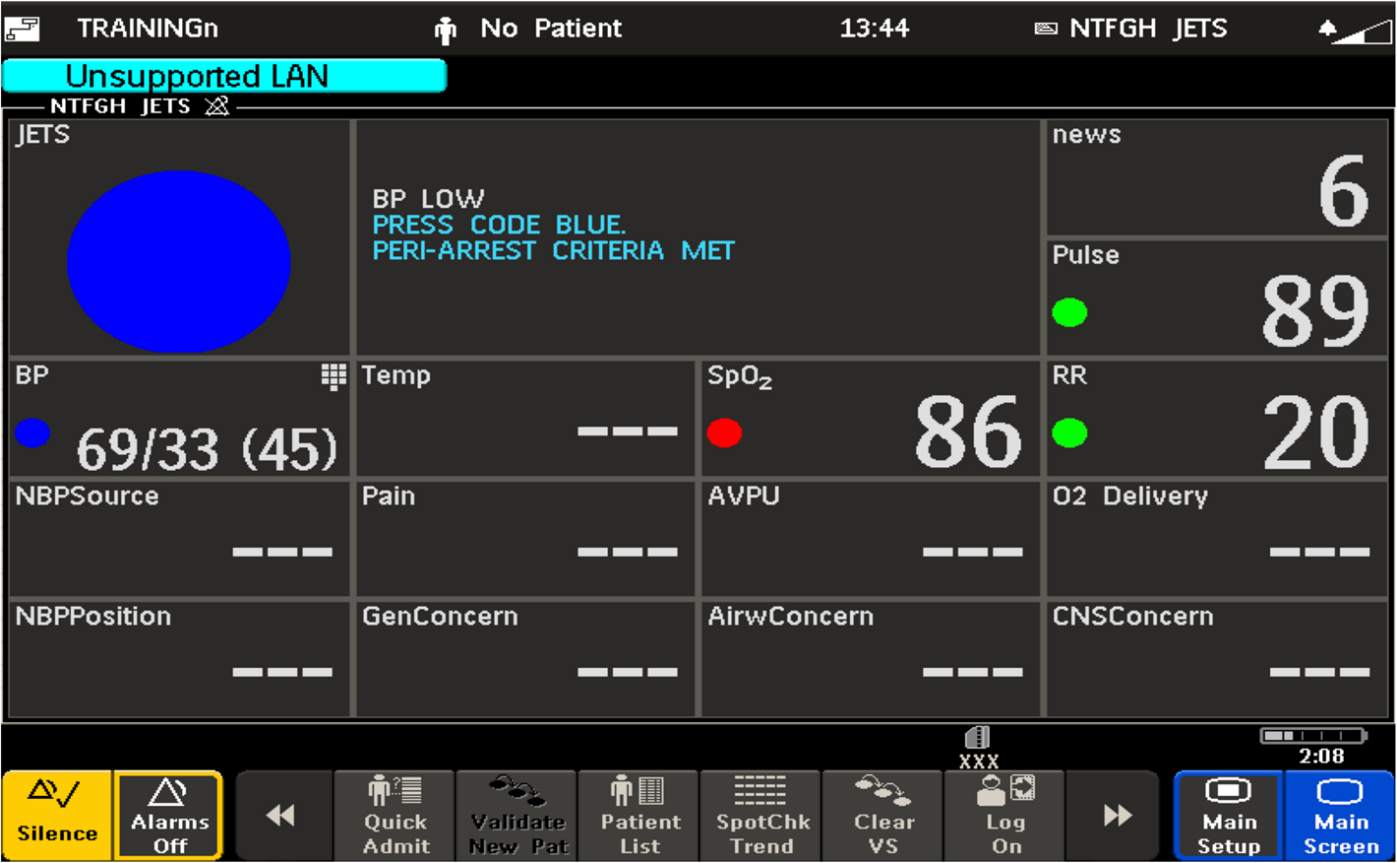
Example of peri-arrest criteria for code blue activation. Patient is identified and verified electronically by the bar code scanner on the mobile Phillips monitor. The vitals to be taken are displayed graphically including heart rate, respiratory rate, blood pressure and oxygen saturation. Vitals are taken at intermittent periods by nurse who confirms that the vitals are accurate by pressing validate which are then sent to the EMR system. At the same time, if the patient’s vitals meet the peri-arrest criteria, there will be a visual alert from the Phillips monitor which alerts the nurse to activate code blue button at bedside

Opened since third quarter of 2015, NTFGH was highly digitalised in patient care with employment of electronic medical records (EMR) system—Epic Systems software (Epic, Verona, WI). Mobile physiological monitors were used to capture patients’ vital signs and send the validated vitals to the EMR system wirelessly. Each monitor consisted of a user interface device, a barcode scanner or input device, parameter processing unit, wireless transmitter to the EMR system and visual and audio alarms. When it was due for vital signs check, patients would be identified and verified electronically by the barcode scanner on the mobile physiological monitor. The vital signs displayed included heart rate (HR), respiratory rate (RR), blood pressure (BP) and peripheral capillary oxygen saturation (SpO_2_) (Fig. 1). Parameters were taken at intermittent periods by nursing staff who confirmed that the parameters were accurate by pressing ‘Validate’, after which, the parameters were sent to the EMR system.

**Fig. 2:**
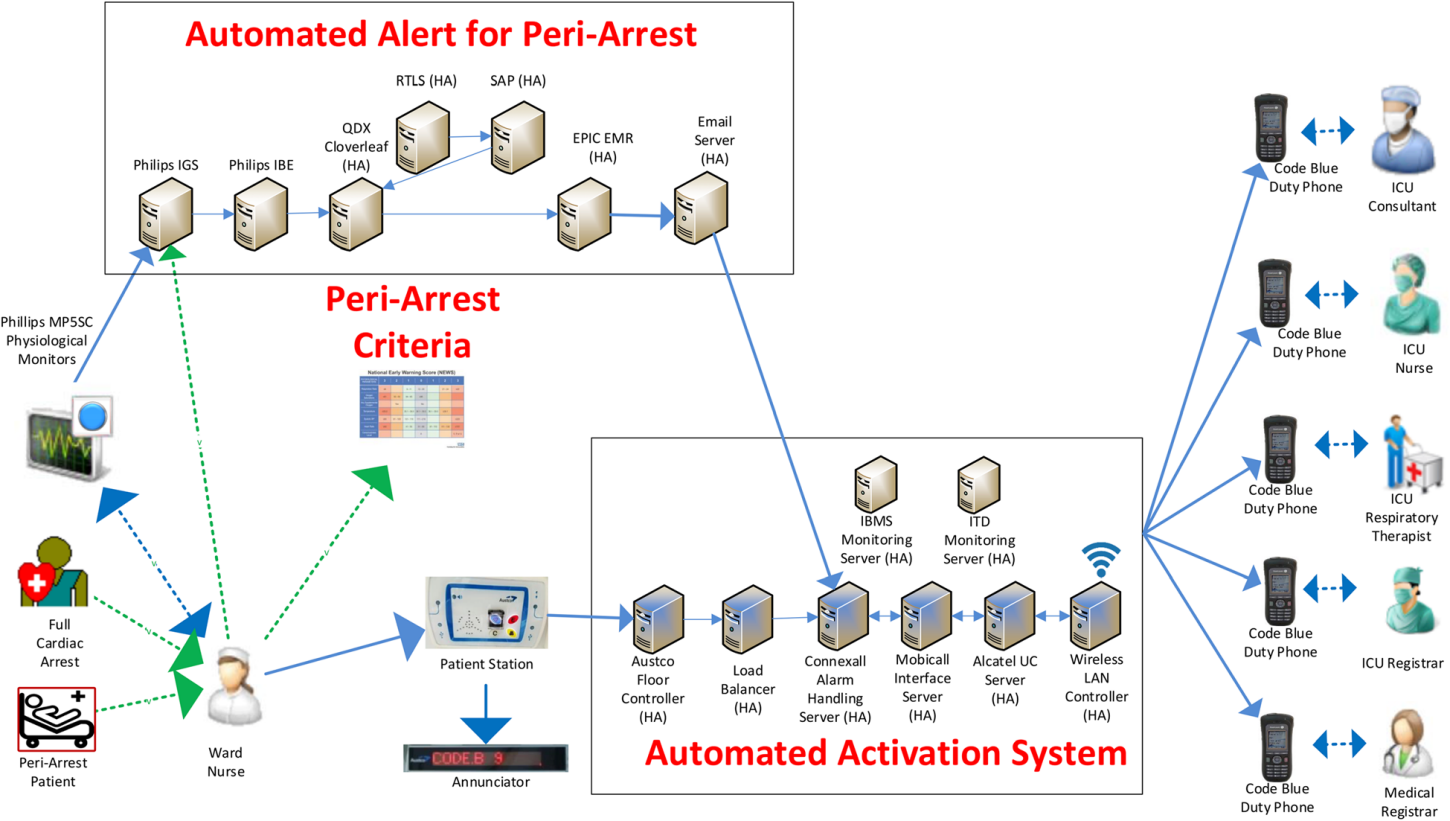
Automated code blue alert and activation system diagram. A patient has a set of vital sign measurement collected by Phillips monitor and validated into EMR system by nurse. Then the vital signs will be further processed through an early warning scoring system configured by a proprietary algorithm in the Phillips and EMR server. If any of the vital sign meets peri-arrest criteria, system will trigger an alert to the code blue phones which contains location of the patient; at the same time, the Philips monitor will give a local audio and visual alarm to remind the ward nurse to press the code blue button at bedside which will activate the Ward Annunciator to display the “Code B” message

In 2018, the ACBAA system based on the peri-arrest criteria was introduced as an extension and supplement to the pre-existing manual code blue activation system. This system aimed to reduce IHCA incidence by automatically alerting trained ICU personnel of critically-ill patients once any of their parameters validated fulfilled requisite peri-arrest criteria, before their condition deteriorated into frank IHCA. The peri-arrest criteria were established based on department consensus as such: airway obstruction requiring immediate intubation; RR < 8 per minute or RR > 45 per minute; SpO_2_ < 80% despite non-rebreathing mask (NRM) 15 liter oxygen per minute; HR < 40 per min or systolic BP < 70 mmHg. Code blue would be automatically activated once any of the vital signs fulfilling the peri-arrest criteria was validated by a nurse in the EMR system (Fig. 2). Even with automated code blue activation, staff would still be prompted by the monitor to press code blue button whenever peri-arrest criteria was met as part of a double security measurement (Fig. 1). Peri-arrest situations from airway obstruction still required the healthcare professional’s clinical recognition and manual activation of code blue. As part of the matrix in the EMR system, the ACBAA system would not be activated if the patient had a “Do Not Resuscitate” order.

### Precautions during COVID-19

As the COVID-19 incidence increased in Singapore in February 2020, our institution implemented enhanced surveillance strategy and strict precaution. In our institution, COVID-19 positive patients and COVID-19 suspect patients who fulfilled criteria predefined by Ministry of Health (MOH) were admitted to the isolation ward with negative-pressure rooms. The official suspect case criteria from MOH generally comprised clinical features (acute respiratory disease) and epidemiologic risk factors. Our institution employed broader screening criteria to minimise the risk of missing COVID-19 cases. All admissions with respiratory symptoms or fever but not fulfilling COVID-19 suspect case criteria would be tested for COVID-19. Those patients would stay in the surveillance wards until COVID-19 PCRs came back negative. The surveillance wards comprised of single rooms with or without negative pressure.

As a precaution, staff working in the isolation ward used full personal protection equipment (PPE) including N95 masks, goggles, disposable caps, gowns and gloves for usual patient care. Powered air-purifying respirator (PAPR), disposable caps, gowns and gloves were used for potential aerosol generating procedures such as suctioning. Staff working in surveillance wards used full PPE for both situations. Surgical masks were mandatory in all other clinical areas. For code blue activation, precautions were taken according to the COVID-19 exposure risk in respective wards as well as procedures performed. For code blue activation in the isolation ward and surveillance ward, PAPR was used for any procedure involving airway management such as bag-mask ventilation, suctioning, intubation and full PPE was used to perform any other procedure such as cardiopulmonary resuscitation (CPR). For code blue activation in wards other than these areas, HCBT used full PPE for airway management and usual standard precaution (surgical mask, apron and disposable gloves) for other management.

### Study design

The precautionary protocols for HCBT response were formally implemented in late February 2020. The study used period from 1 March 2020 to 31 May 2020, as the COVID-19 study period and the period from 1 March 2019 to 31 May 2019, as the pre-COVID-19 control period. The inclusion criteria were patients who had a code blue activation during their inpatient stay. Patients who were younger than 21 years old or had a “Do Not Resuscitate” order were excluded. All false code blue activations were excluded, such as accidentally pressing on the code blue button or wrongly validated vital signs by staff which triggered the AABCA system. The study retrospectively collected data of all the patients included in the study. The HCBT response time for code blue activation in our institution was calculated as the time from code blue phone alert to time of arrival of HCBT at patient’s bedside.

The study was approved by Institutional Review Board (IRB) with waiver of consent from Singapore’s National Healthcare Group Domain Specific Review Board (DSRB 2020/00861).

### Outcome measurement

The primary outcome was IHCA survival rate to hospital discharge. The secondary outcomes included incidence of code blue activation per 1000 hospital admissions, incidence of peri-arrest code blue activation per 1000 hospital admissions, incidence of IHCA per 1000 hospital admissions; the rate of return of spontaneous circulation (ROSC) for IHCA and the rate of survival to hospital discharge of all code blue activation and peri-arrest code blue activation. ROSC was defined as the onset of an organised rhythm with a palpable pulse and a measurable blood pressure for at least 30 s [11].

### Statistical analysis

Continuous data was reported as means and 95% confidence intervals. Categorical variables were reported as numbers and percentages of patients in each category. Proportions were compared using Chi-square or Fisher exact tests and continuous variables were compared using the t test or Wilcoxon rank-sum test. For outcome analysis, the odds ratio (OR) and 95% confidence interval were calculated. Data was analysed using SPSS statistical software version 13.0. Two-tailed *P* values < 0.05 were considered significant.

## Results

There were 9130 hospital admissions in the COVID-19 study period and 9725 hospital admissions in the pre-COVID-19 control period (Table 1). Despite a 6% decrease in hospital admissions during COVID-19 study periods as compared to the pre-COVID-19 control period, the number of code blue activations increased by 35% (34 cases increased to 46 cases) (Table 1). The baseline characteristics of the included patients are shown in Table 2. Patients in the COVID-19 cohort were younger whereas more patients in the pre-COVID-19 cohort had history of malignancy. The other baseline characteristics such as gender, hypertension, diabetes, ischaemic heart disease, chronic liver disease, chronic lung disease and chronic kidney disease were similar between two periods. The Acute Physiology And Chronic Health Evaluation (APACHE) II score calculated within 24 h of ICU admission was similar between two periods. In the COVID-19 period, the average HCBT response time was 3.50 min, which was significantly longer as compared to pre-COVID-19 period of average 2.45 min (Table 2).

**Table 1:** Code blue activation summary

	Pre-COVID-19 period	COVID-19 period	OR
Total hospital admission	9725	9130	
Total code blue activation	34	46	1.44 (0.91-2.32)
Peri-arrest	24	29	1.28 (0.71-2.31)
In-hospital cardiac arrest	10	17	1.81 (0.78-4.41)

*OR* odds ratio

**Table 2:** Baseline characteristics

	Pre-COVID-19 period (n = 34)	COVID-19 period (n = 46)	P value
Age (year)	70.8 ± 12.7	65.0 ± 12.8	0.04
Male gender (%)	23 (64.8)	28 (66.4)	0.42
DM (%)	24 (68.9)	28 (65.5)	0.74
Hypertension (%)	17 (54.4)	22 (48.7)	0.27
IHD (%)	10 (24.8)	10 (28.6)	0.39
CVA (%)	8 (18.1)	12 (16.8)	0.85
Chronic kidney disease (%)	9 (23.2)	13 (31.1)	0.93
Chronic lung disease (%)	5 (12.0)	7 (14.3)	0.99
Chronic liver disease (%)	1 (8.0)	1 (5.8)	0.81
Malignancy (%)	6 (14.4)	1 (8.4)	0.01
APACHE II Score	29.1 ± 10.1	29.3 ± 9.2	0.93
HCBT response time (min)	2.45 ± 1.25	3.50 ± 2.12	0.01

*DM* diabetes mellitus, *IHD* ischaemic heart disease, *CVA* cerebrovascular accident, *APACHE II* Acute Physiology And Chronic Health Evaluation II, *HCBT* hospital code blue team

**Fig. 3:**
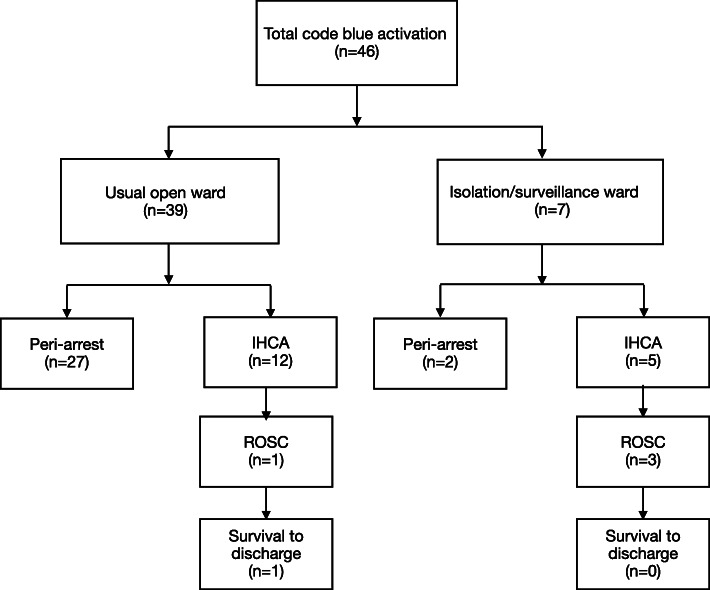
Flow diagram of COVID-19 study period code blue activation details. IHCA, in-hospital cardiac arrest; ROSC, return of spontaneous circulation

Figure 3 was a flow diagram of code blue activations in wards with respective precautions during COVID-19 period. There were a total of 7 code blue activations in the isolation and surveillance wards and 39 code blue activation in wards without COVID-19 positive or suspect case (open wards). For the pre-COVID-19 period, no code blue activation happened in the isolation ward which was mainly for tuberculosis cases or other airborne precaution cases in our institution.

**Fig. 4:**
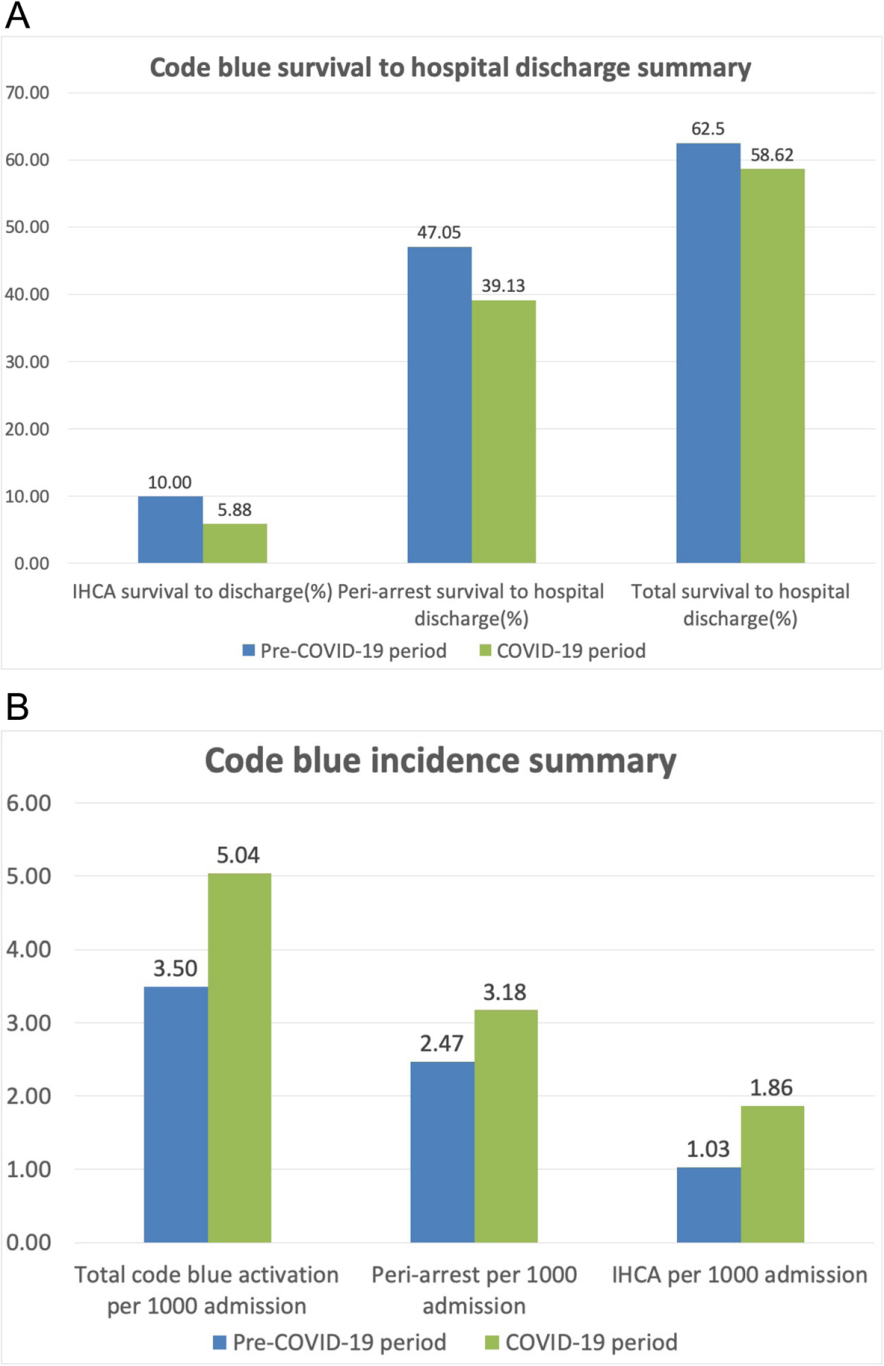
Primary and secondary outcomes summary. IHCA, in-hospital cardiac arrest

In terms of primary outcome, the rate of survival to hospital discharge for IHCA was 5.88% in the COVID-19 period as compared to 10.0% in the pre-COVID-19 period [odds ratio (OR), 0.72; 95% confidence interval (CI), 0.26-1.95] (Table 3, Fig. 4a). For secondary outcomes, there were more total code blue activations per 1000 hospital admissions in the COVID-19 period (OR, 1.44; 95% CI, 0.91-2.32); more code blue activations for peri-arrest per 1000 hospital admissions in the COVID-19 period (OR, 1.28; 95% CI, 0.71-2.31); as well as more code blue activations for IHCA per 1000 hospital admissions in the COVID-19 period (OR, 1.81; 95% CI, 0.78-4.41) (Table 1, Fig. 4b). ROSC rate of IHCA in the COVID-19 period was 58.82%, lower as compared to 90% in the pre-COVID-19 period (OR, 0.35; 95% CI, 0.03-2.76) (Table 3). The rates of survival to hospital discharge for all code blue activations and peri-arrest code blue activation were also lower in the COVID-19 period (all code blue activations: OR, 0.72; 95% CI, 0.26-1.95; peri-arrest code blue activation: OR, 0.85; 95% CI, 0.24-2.94) (Table 1, Fig. 4a). Table 4 lists the reasons of code blue activation for peri-arrest situation.

**Table 3:** Code blue activation outcome

	Pre-COVID-19 period (n = 34)	COVID-19 period (n = 46)	P value
Initial shockable rhythm	1 (n = 10)	3 (n = 17)	1.92 (0.12-112.3)
ROSC	9 (n = 10)	10 (n = 17)	0.35 (0.03-2.76)
Total survival to hospital discharge	16 (n = 34)	18 (n = 46)	0.72 (0.26-1.95)
Peri-arrest survival to hospital discharge	12 (n = 24)	17 (n = 29)	0.85 (0.24-2.94)
Cardiac arrest survival to discharge	1 (n = 10)	1 (n = 17)	0.56 (0.01-49.01)

*OR* odds ratio, *ROSC* return of spontaneous circulation

**Table 4:** Reason for peri-arrest code blue activation

Peri-arrest cause	Pre-COVID-19 period (n = 24)	COVID-19 period (n = 29)
Airway obstruction (%)	0 (0)	1* (3.4)
RR (%)	0 (0)	2 (6.8)
SpO_2_ (%)	9 (37.5)	6 (20.7)
HR (%)	3 (12.5)	1 (3.4)
SBP (%)	5 (20.8)	12 (41.4)
Others (%)	7^#^ (29.2)	7^#^ (24.1)

*RR* respiratory rate, *SpO*_*2*_ peripheral capillary oxygen saturation, *HR* heart rate, *SBP* systolic blood pressure

*Code blue activated for massive bleeding from tracheostomy site

#Code blue activated for acute drop in Glascow Coma Score with concerns of airway patency

## Discussion

To the authors’ knowledge, this is one of the first few studies to describe the incidence and outcomes of IHCA during COVID-19 pandemic. Whilst the recent study about IHCA done by Miles et al. also observed decreased survival rate of IHCA during the COVID-19 pandemic as compared to pre-COVID-19 pandemic, 79% of the IHCA patients in their study were COVID-19 positive, which was a major difference as compared to our study [9]. For all code blue activation during COVID-19 period in our study, only one IHCA case was later confirmed as COVID-19 positive. Hence, the increased incidence and decreased survival rate of IHCA and total code blue activation observed in our institution is unlikely directly caused by COVID-19 disease process.

COVID-19 has imposed a strong demand on the public healthcare system with the result of resource redistribution and reprioritisation to minimise COVID-19 transmission risk. Since late February 2020, as part of COVID-19 preparedness, the inpatient facilities in our institution were reorganised and adapted to ever changing circumstances. A number of rehabilitation wards in Jurong Community Hospital (JCH), a subacute hospital linked to NTFGH via a link bridge, were converted to acute care wards to admit acute non-COVID patients, a common resource reallocation strategy nationwide in pandemic [12, 13]. Whilst the medical resources are equally shared between NTFGH and JCH, the staff in JCH who usually care for subacute patients now have to care for acute patients. There might be a possibility of unfamiliarity with the acute patient profile resulting in missing a deteriorating patient. Also due to the precautions taken against COVID-19, there would be some inevitable delay in attending to a deteriorating patient. Particularly for patients admitted to surveillance and isolation wards, the deterioration of patients who cannot call for help themselves might not be caught until staff attends to them as they are all in single rooms, by when the patients could have deteriorated into peri-arrest or cardiac arrest situation. Based on above, we cautiously attribute the increased IHCA incidence during COVID-19 pandemic in our institution due to diversion of resources and manpower to care for COVID-19 patients as well as prevent COVID-19 transmission.

It is expected the survival of in-hospital cardiac arrest (IHCA) during COVID-19 pandemic will be lower due to precautions taken before initiating resuscitations [14]. Our study did observe a decreased rate of IHCA survival to home discharge. The response time of HCBT during COVID-19 period is significantly longer than pre-COVID-19 period, which could be associated with the decreased survival rate. Whilst precautions taken have minimised the risk of healthcare providers’ exposure to COVID-19, donning PPE could be time consuming and may cause delay in attendance to patients with IHCA. Performance and behavioural change in response to IHCA may have impacted on HCBT response as well [15]. Mask ventilation and tracheal intubation are considered aerosol-generating procedures, however it is still uncertain whether chest compression or defibrillation are aerosol generating procedures due to very limited evidence [16, 17]. For IHCA patients with suspected or confirmed COVID-19 disease, European Resuscitation Council (ERC) COVID-19 Guidelines recommends use of airborne-precautions for aerosol-generating procedures (chest compressions, airway and ventilation interventions) during resuscitation [18]. The minimum airborne-precaution PPE is defined as gloves; long-sleeved gown; filtering face piece 3 (FFP3) or N99 mask/respirator (FFP2 or N95 if FFP3 not available) and eye and face protection (full-face shield/visor or polycarbonate safety glasses or equivalent). Alternatively, PAPRs with hoods may be used [18]. The precautionary measure used by our HCBT is in accordance with the ERC COVID-19 guidelines.

Whilst it is imperative for code blue responders to take appropriate precautions during the COVID-19 pandemic, we should also make every effort to reduce the incidence and improve the outcome of IHCA. Our institutional experience has demonstrated that the code blue activation system with peri-arrest criteria might have a role in reducing IHCA incidence [5]. In our institution, the incidence of IHCA was 1.64 per 1000 hospital admissions before ACBAA system implementation; and has dropped down to 0.88 per 1000 hospital admissions after ACBAA system [5]. Although the IHCA incidence per 1000 hospital admissions during COVID-19 period has increased to 1.88, it is still close to pre-pandemic international incidence of 1.6 to 10.16 per 1000 hospital admissions. The use of automated physiological monitors to assist in the acquisition of vital signs and identifying deteriorating patients could have a unique advantage in the pandemic situation [19]. With regards to measures to improve IHCA resuscitation outcomes, we propose full PPEs readily available for code blue responders in the emergency trolley and regular simulation of code blue activation with airborne precautions.

The study has a few limitations. Firstly, due to the single-centre, retrospective and observational nature of the study, other confounders may have contributed to the trend of increased IHCA incidence and reduction in survival rate. Secondly, none of the results were statistically significant though there was a consistent trend of more IHCA and decreased survival rate of IHCA. This could be due to overall low incidence of IHCA in our institution and relative short study period.

## Conclusions

Our study observed a trend towards higher incidence and lower rate of survival to hospital discharge for IHCA during COVID-19 pandemic. This is likely indirectly related to healthcare resource constrain and precautions taken to reduce nosocomial transmission during pandemic, rather than directly related to COVID-19 disease given the relative low COVID-19 incidence and mortality in Singapore. This reminds us that in a pandemic situation, whilst mobilising and maximising available resources to reduce nosocomial transmission risk is undoubtfully important; it is equally important to implement strategies to ensure the quality of care for acutely ill non-COVID-19 patients.

## Data Availability

The datasets generated during and/or analysed during the current study are not publicly available due to confidentiality but are available from the corresponding author on reasonable request.
